# The Effect of Process Parameters on the Microstructure, Stability, and Sensorial Properties of an Emulsion Cream Formulation

**DOI:** 10.3390/pharmaceutics16060773

**Published:** 2024-06-06

**Authors:** Pui Shan Chow, Ron Tau Yee Lim, Febin Cyriac, Jaymin C. Shah, Abu Zayed Md Badruddoza, Thean Yeoh, Chetan Kantilal Yagnik, Xin Yi Tee, Annie Bao Hua Wong, Vernissa Dilys Chia, Guan Wang

**Affiliations:** 1 Institute of Sustainability for Chemicals, Energy and Environment, Agency for Science, Technology and Research (A*STAR), 1 Pesek Road, Jurong Island, Singapore 627833, Singapore; ann_chow@isce2.a-star.edu.sg (P.S.C.);; 2 Pfizer Inc., Groton, CT 06340, USA; abuzayedmd.badruddoza@pfizer.com (A.Z.M.B.);

**Keywords:** emulsion, cream, topical formulation, microstructure, rheology, sensorial, manufacturing process, design of experiment

## Abstract

A classical emulsion formulation based on petrolatum and mineral oil as the internal phase with emulsifier wax as a typical topical emulsion cream was investigated for the effect of process parameters on drug product quality and performance attributes. The Initial Design of Experiment (DoE) suggested that an oil phase above 15%, coupled with less than 10% emulsifying wax, resulted in less stable emulsions. Different processing parameters such as homogenization speed, duration, cooling rate, and final temperature showed minimal influence on properties and failed to improve stability. The final DoE suggested that the optimal emulsion stability was achieved by introducing a holding period midway through the cooling stage after solvent addition. Within the studied holding temperature range (25–35 °C), a higher holding temperature correlated with increased emulsion stability. However, the application of shear during the holding period, using a paddle mixer, adversely affected stability by disrupting the emulsion microstructure. IVRT studies revealed that the release of lidocaine was higher in the most stable emulsion produced at a holding temperature of 35 °C compared to the least stable emulsion produced at a holding temperature of 25 °C. This suggests that a holding temperature of 35 °C improves both the stability and active release performance. It appears that a slightly higher holding temperature, 35 °C, allows a more flexible and stable emulsifying agent film around the droplets facilitating stabilization of the emulsion. This study offers valuable insights into the relationship between process parameters at various stages of manufacture, microstructure, and various quality attributes of emulsion cream systems. The knowledge gained will facilitate improved design and optimization of robust manufacturing processes, ensuring the production of the formulations with the desired critical quality attributes.

## 1. Introduction

Topical semisolid dosages such as creams, ointments, and gels are an important class of drug delivery systems that are typically used for local treatment of various kinds of skin diseases and disorders. Emulsions (either oil-in-water (O/W) or water-in-oil (W/O)) which have been frequently formulated into different semisolid dosage forms are gaining significant importance because of their many advantages. Emulsions offer the ability to solubilize both lipophilic and hydrophilic drugs, ease of application, and patient acceptability [1,2]. Despite their remarkable benefits, a high degree of complexity and variability is associated with the emulsion’s microstructure. Typically, an emulsion semisolid dosage form is a complex multiphase system in which various microstructures (arrangement of matter and state of aggregation) may exist. The microstructure of an emulsion semisolid dosage can be determined by how the components are employed in its formulation (e.g., size and size distribution of the dispersed phase droplets, size and shape of crystalline materials, polymorphism, partition coefficient of the active substance between the different phases, rheology of the systems, etc.) [3,4].

When developing emulsion-based semisolid formulations, it is critical to understand and control their microstructural characteristics since the microstructure is greatly related not only to the organoleptic properties of the product but also to the product shelf-life, quality, and performance attributes [5,6]. Dispersed phase droplet or globule size is one of the critical quality attributes of the emulsion-based formulations which could be controlled during the manufacture by regulating the formulation parameters, as well as processing conditions such as homogenization speed and time, temperature, and order of mixing [7]. The droplet microstructure due to its size and distribution contributes not only to the physical stability of topical semisolid products but also to the product quality and performance such as uniformity, texture and rheology attributes, spreadability, sensory properties (e.g., skin feel, adhesion), drug release, and skin permeation. Rheological behavior can provide valuable insights into the internal microstructure of formulations which is strongly correlated with their physical stability. Though ensuring the physical stability by tuning the microstructure is critical, it remains a key formulation design challenge. Therefore, understanding and assessing various formulation and manufacturing processing factors that determine the microstructure could be useful to obtain the long-term physical stability, as well as the desired physicochemical properties and performance characteristics, of the drug product.

The manufacture of emulsion-based cream dosages involves several operation steps including a series of heating, cooling, and mixing steps. The mixing of multiphasic highly viscous (non-Newtonian) systems poses more challenges compared to less viscous single-phase systems [8]. The conventional semisolid O/W creams often use petrolatum and wax-like materials such as emulsifying wax and cetostearyl alcohol that require high temperature to be melted. Use of different mechanical mixers such as central agitator, scraper and homogenizer (shear), and appropriate processing conditions (e.g., temperature, mixing time) can provide better heat transfer and mixing of various excipients to ensure the homogeneity and targeted droplet size of the internal phase [7]. The homogenizer can disrupt the dispersed phase and break down the emulsion droplets or globules into smaller ones utilizing high shear forces [9]. Uncontrolled levels of homogenization time, temperature, and the extent of shear rate could induce phase separation resulting from various de-mixing phenomena, thus impacting the physical stability of emulsion creams [10,11]. The cooling process can also influence the crystallization and solidification behaviors of lipid-based stabilizers, such as mono- and di-glycerides and petrolatum, which in turn impact the internal structure and stability of emulsion-based ointments and creams [12,13]. Thus, the processing parameters involved in each manufacturing step need to be carefully controlled and optimized during manufacturing of emulsion semisolid dosages. The objective of this study was to understand the influence of typical manufacturing processing parameters on the microstructure, stability, and sensorial properties of an O/W topical cream formulation through a systematic DoE approach, with the ultimate goal of designing a robust manufacturing process for delivering stable O/W semisolid creams with the desired microstructure, properties, and performance attributes.

## 2. Materials and Methods

### 2.1. Materials

PEG400 was purchased from PanReac AppliChem (Darmstadt, Germany), glyceryl monostearate (GMS) was purchased from Spectrum (Palisades Park, NJ, USA), light mineral oil was purchased from ThermoFisher Scientific (Loughborough, UK), and white petrolatum (WP) was supplied by H&R Gruppe (Hamburg, Germany). Oleyl alcohol, Tween 20, cetyl alcohol, and stearyl alcohol were supplied from BASF (Monheim, Germany). All the ingredients were compendial grade except for glyceryl monostearate which was food grade. The active pharmaceutical ingredient, Lidocaine, was purchased from Sigma Aldrich (Singapore). Ultra-pure water (Milli-Q^®^ Gradient A10^®^, Millipore, Malsheim, France) was used. All the ingredients were used directly as supplied for emulsion cream preparation.

### 2.2. Emulsion Cream Preparation

For the DoE studies to understand the effect of formulation composition and processing on attributes of the formulation, initially, a range of compositions were investigated. Table 1 shows the range of compositions of emulsion creams used in this study. The creams consisted of purified water and PEG400 as the aqueous phase and light mineral oil and white petrolatum as the oil phase. Emulsifying wax as 5–10 wt% of the total formulation was made up of 33 wt% glyceryl monostearate, 14 wt% Tween 20, 33% cetyl alcohol (CA), and 20 wt% stearyl alcohol (SA). The emulsifying wax served as the primary and oleyl alcohol served as secondary emulsifying agents for stabilizing the o/w emulsion cream.

**Figure 1:**
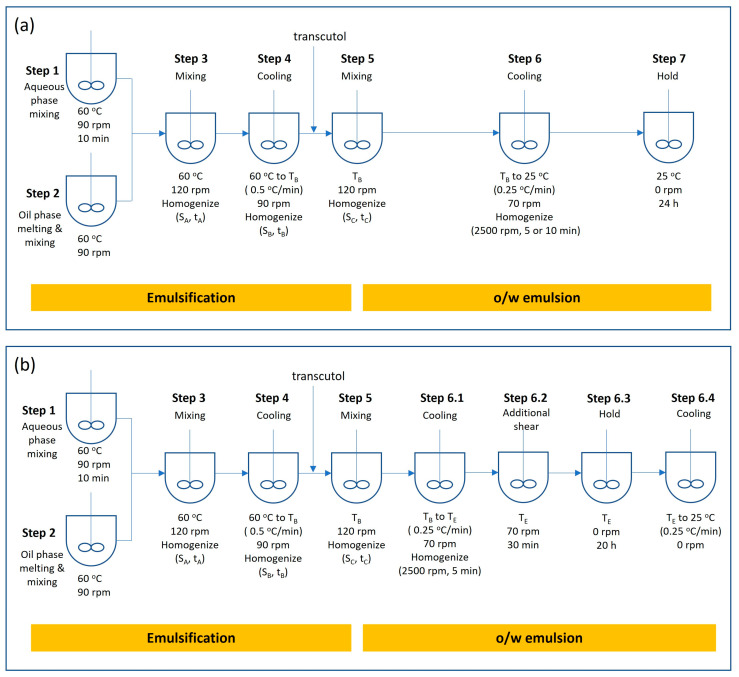
(**a**) Original process flow for preliminary experiments. (**b**) Modified process flow for final DoE. S denotes homogenization speed, t denotes homogenization duration, T denotes target temperature.

A high-speed mixing emulsifying equipment (ESCO EL1 ECO, ESCO-Labor AG, Switzerland), coupled with a Julabo FP50 water circulator, was used to prepare the emulsion cream as shown in Figure 1. The emulsion cream preparation steps are outlined below in more details. The levels of each processing condition are listed in Table 2.

Step 1. Charge purified water and PEG 400 into the main vessel, and heat to 60 ± 5 °C and mix at 90 rpm for 10 min.
Step 2. Charge light mineral oil, white petrolatum, emulsifying wax, and oleyl alcohol in an appropriately sized jacketed vessel. Heat the content to 60 ± 5 °C, and mix at 90 rpm until all materials are fully melted and uniformly mixed.
Step 3. Transfer the content of the jacketed vessel into the main vessel. Homogenize at speed S_A_ for a duration of t_A_, and mix at 120 rpm at 60 ± 3 °C.
Step 4. Cool the content of the main vessel at 0.5 ± 0.1 °C/min while mixing at 90 rpm and homogenizing at speed S_B_ rpm for t_B_ minutes until the temperature reaches T_B_.
Step 5. Dissolve lidocaine in transcutol at T_B_. Charge transcutol into the main vessel while mixing at 120 rpm. After the addition is completed, homogenize at speed S_C_ rpm for t_C_ minutes at T_B_.
Step 6. Cool the content to 25 ± 3 °C at 0.25 ± 0.1 °C/min while mixing at 70 rpm. Once the temperature reaches 25 °C, homogenize at 2500 rpm for 5 or 10 min.
Step 7. Stop mixing, and keep the product stored at 25 °C for 24 h.

Based on the results of the above DoE, in the final DoE, the process was modified to apply additional shear to the emulsion cream during the cooling process after transcutol addition. Steps 1 to 5 are identical to the original process. The cooling step 6 of the original process was paused halfway where shear was applied for 30 min, followed by a holding period of 20 h before the mixture was finally cooled to 25 °C. The steps after step 5 of the modified process are described below.

Step 6.1. Cool the content to temperature T_E_ at 0.25 ± 0.1 °C/min while mixing at 70 rpm and homogenizing at 2500 rpm for 5 min.
Step 6.2. Mix at 70 rpm at temperature T_E_ for 30 min (additional shear step).
Step 6.3. Hold at temperature T_E_ for 20 h with no mixing.
Step 6.4. Cool at 0.25 ± 0.1 °C/min to 25 ± 3 °C (not required if T_E_ is set at 25 °C).

### 2.3. Characterization Techniques

#### 2.3.1. Microscopy Analysis

Microscopic images of the emulsion cream were acquired using an Olympus BX51 polarizing microscope (Olympus, Singapore) equipped with a Nikon DS-Fi3 high resolution camera (Nikon, Singapore). The images were analyzed using the NIS-Elements AR imaging analysis software version 4.60.00 (Nikon, Singapore). The size of the emulsion droplets was measured manually. At least 450 droplets were measured to ensure statistical significance and representative of the bulk product. Since the emulsion cream is an o/w emulsion, the droplets observed are the oil droplets.

#### 2.3.2. Physical Stability

The physical stability of emulsion cream samples processed with different processing parameters were characterized using a LUMiSizer (LUM GmbH, Berlin, Germany). It accelerates the creaming or settling in the samples due to the inherent instability of emulsions with the use of centrifugal force [14,15]. The light passing through the sample was monitored continuously in terms of both time and position across the entire sample length while subjecting the sample to centrifugation, aiming to hasten phase separation. The rate at which phases separate relative to each other served as an indicator of the sample’s physical instability. The instability analysis was performed using the SepView6.0 software (LUM GmbH, Berlin, Germany). An instability index (IS) was determined by integrating the normalized transmitted light over the entire length of the sample [16]. The instability index ranges from 0 to 1, with 0 denoting a stable system and 1 an unstable system [17]. During centrifugation, an unstable system will undergo phase separation, leading to the emergence of a clearer phase that allows more light to transmit through the sample. Consequently, this results in a higher instability index. Therefore, a higher instability index signifies a greater degree of phase separation and, consequently, lower stability. The emulsion cream sample (~0.4–0.5 mL) was placed in a LUMisizer polycarbonate cell (2 mm) (LUM GmbH, Berlin, Germany) and subjected to 4000 rpm at 25 °C for a measurement duration of 7.5 h.

#### 2.3.3. Rheological Properties

The rheological characterization of emulsion cream samples was conducted using an MCR 302 rheometer (Anton Paar, Graz, Austria). All measurements were carried out at 25 ± 0.1 °C utilizing sandblasted steel parallel plates (diameter: 25 mm, surface roughness: Ra = 5.4 µm) with a measurement gap of 1 mm. After adjusting the gap to 1 mm, a rest period of 10 min was allowed to relieve residual stress and facilitate the formation of the microstructure. Small Amplitude Oscillatory Shear (SAOS) measurements were performed at 1 Hz frequency, spanning a strain range from 0.001 to 1000%, without imposing any time constraints on the measurement duration. Linear viscoelastic properties such as storage modulus (G′), loss modulus (G″), and complex viscosity (η*) were derived from the SAOS measurements.

#### 2.3.4. Sensorial Properties—Texture and Tribology Analysis

A Brookfield CTX texture analyser (AMETEK Brookfield, Middleboro, MA, USA) was used to conduct texture analysis. Penetration tests on the samples (50 mL) were performed using a steel hemispherical probe of 12.7 mm diameter [18]. During the measurements, the probe penetrated the sample over a depth of 20 mm at a pre-set load and remained at that position for about 1 s before retracting to its initial position. The downward and upward movements of the probe were controlled at a speed of 0.25 mm/s. The data were analyzed using TEXTURE PRO version 1.0 build 19 (AMETEK Brookfield, Middleboro, MA, USA) to obtain different textural attributes. The measurements were repeated thrice to check reproducibility. The readers are referred to Chow et al. [6] for methodology for obtaining firmness, adhesiveness, and stringiness from the textural analysis.

Tribological measurements were conducted using an in-house linear reciprocating tribometer on Bioskin (Beaulax, Co Ltd., Saitama, Japan), an artificial skin composed of urethane elastomer designed to mimic human skin characteristics. Bioskin was utilized to minimize variations inherent in actual skin and ensure consistent measurements. Frictional resistance of different creams was assessed at 25 ± 0.5 °C and RH 55 ± 3% by sliding a 25 mm diameter hemispherical PEEK probe against a Bioskin plate at a speed of 10 mm/s and a load of 0.5 N. Approximately 0.4 g of sample was used for each measurement, and the total displacement of the probe was 30 mm. At least three measurements were performed for each sample. For further details on the methodology, readers are referred to Cyriac et al. [18,19].

#### 2.3.5. Thermal Analysis

Digital scanning calorimetry thermograms were acquired using a Mettler Toledo DSC3 calorimeter (Mettler Toledo, Singapore). The instrument was calibrated using indium as standards prior to measurements. About 5 mg of sample was placed in a crimpled aluminum pan and heated at a rate of 1 °C/min from 15 to 65 °C after equilibrating at 15 °C for 10 min and subsequently cooled to 15 °C at the same rate. Two cycles of measurements were performed for each sample (heating—cooling—heating—cooling). The glass transition temperature (T_g_), melting temperature (T_m_), and melt crystallization temperature (T_c_) were determined using STARe SW V14.00 analysis software, version 14.00. T_g_ was determined as the midpoint of the change in heat capacity of the sample, while T_m_ and T_c_ were determined as the onset temperatures. The samples were purged with a stream of nitrogen at 50 mL/min.

#### 2.3.6. High Performance Liquid Chromatography (HPLC) Assay of Lidocaine

The assay of lidocaine was analyzed using an HPLC (1100 series, Agilent Technologies, Santa Clara, CA, USA) equipped with a ZORBAX Eclipse Plus C18 column (4.6 mm × 250 mm, 5 µm) (Agilent Technologies, Santa Clara, CA, USA) maintained at 25 °C. A mobile phase consisting of a mixture of acetonitrile and water (20:80 *v*/*v*) and 5% acetic acid was used at a flow rate of 1 mL/min. The injection volume was 20 µL, with a retention time ranging from 5.1 to 5.3 min, and detection was performed at a wavelength of 262 nm.

#### 2.3.7. In Vitro Release

In vitro release testing (IVRT) (membrane permeation) studies from the emulsion creams containing 1 wt% of lidocaine were carried out using a Phoenix RDS automated diffusion testing system (Teledyne Hanson, Chatsworth, CA, USA). This system comprises a 6-cell dry heat block and an automated sampling mechanism, enabling simultaneous in vitro release experiments on 6 samples. Each 14 mL Franz cell was filled with a receptor medium consisting of 70:30 *v*/*v* ethanol/water solution to ensure that the solubility of lidocaine is sufficiently high to maintain sink condition. The receptor medium was maintained at 32 ± 1 °C by the dry heat block and continuously stirred at 200 rpm. Approximately 0.25 g of emulsion cream was uniformly spread on a dosage chamber with a 0.45 µm nylon membrane (25 mm disc) placed between the dosage chamber and receptor medium for in vitro release experiments. Nylon membrane was selected for this study due to its inert nature, as well as its minimal capacity for absorbing lidocaine [20]. The cell was sealed by placing a glass cover on top of the dosage chamber. Aliquots of 0.2 mL sample were withdrawn from the cell receptor compartment through the sampling port at 1 h intervals and analyzed for their concentrations using the HPLC method described previously.

## 3. Results and Discussion

### 3.1. Preliminary Experiments

The complexity of the manufacturing process, characterized by numerous processing parameters, necessitated the execution of several sequential Design of Experiment (DoE) studies. These studies were essential to refine the range of processing parameters that would have significant effects on the emulsion cream. Subsequently, a more detailed DoE was conducted on specific stages after evaluating the preliminary experimental data.

The goal of the first set of experiments was to identify a suitable composition for subsequent DoE studies. Runs EM-D1 to EM-D4 (see Table 3) were conducted to investigate the effect of emulsion cream compositions by varying the emulsifying wax and mineral oil contents. Both EM-D1 and EM-D2 have the lowest emulsifying wax content (5 wt%), whereas the mineral oil contents of EM-D1 and EM-D2 were 5 wt% and 10 wt%, respectively. EM-D3 and EM-D4 have the highest emulsifying wax content (10 wt%), whereas the mineral oil contents of EM-D3 and EM-D4 were 5 wt% and 10 wt%, respectively. The homogenization speed [S_A_] was set at 8000 rpm and applied for 10 min, while the cooling rate was set at 0.5 °C/min and transcutol addition temperature [T_B_] was set at 43 °C. The homogenization speeds [S_B_] and [S_C_] were set at 2500 rpm. No significant temperature increase was observed in the mixture within the main vessel, even when employing the highest homogenization speed and longest duration tested in this study.

**Figure 2:**
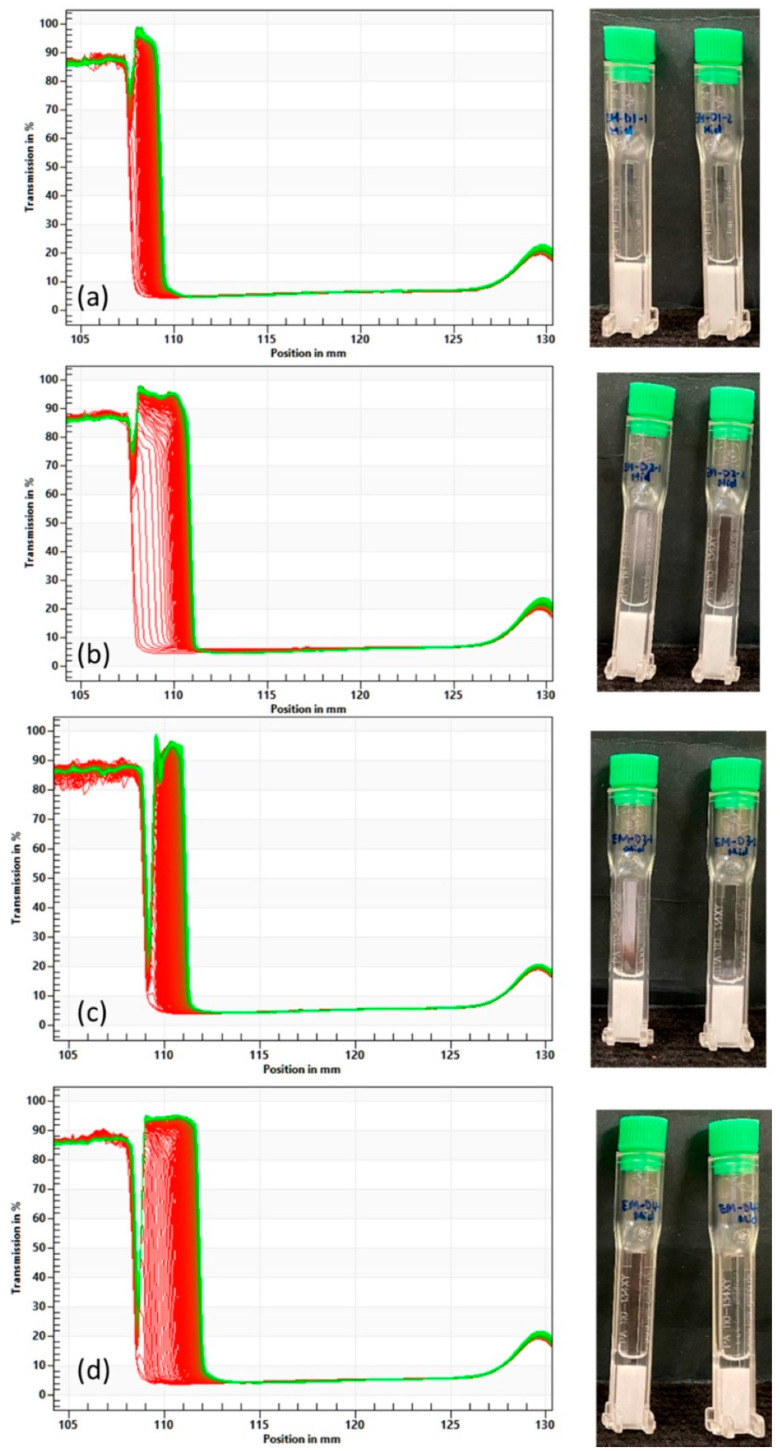
Evolution of light transmission with time vs. length of the sample (**a**) EM-D1, (**b**) EM-D2, (**c**) EM-D3, and (**d**) EM-D4, measured by the Lumisizer and the corresponding photographs of the samples after measurement. The *y*-axis of the graph represents the % light transmission, and *x*-axis represents the vertical position in mm. In each graph, red lines are the transmission data of the first and subsequent scans, while the green line is the transmission data of the last scan.

Figure 2 shows the evolution of light transmission with time for EM-D1 to EM-D4 measured by the Lumisizer together with the corresponding photographs of the samples after measurement. The transmission profiles showed that bleeding (syneresis) [1,13] was observed on the top part of all the four emulsion creams (EM-D1 to EM-D4). A larger amount of syneresis was observed for EM-D2 and EM-D4, while the extents of syneresis for EM-D1 and EM-D3 were comparable. This suggests that both EM-D2 and EM-D4 were less stable than EM-D1 and EM-D3, which is also reflected by their respective instability index in Table 3. The poor stability of EM-D2 and EM-D4 could be attributed to the high mineral oil content (10%). Due to the high centrifugal force used in the Lumisizer, the samples inevitably phase separate to different extents. In order to obtain a more realistic indication of the stability of the samples under normal gravitational force, bottle tests were also performed at accelerated aging condition of 40 °C/75% RH. Figure S1 (Supplementary Information) shows that EM-D3 remained stable for 6 days (the aging stability monitoring did not continue after 6 days as it was not the main characterization test) but EM-D1, EM-D2 and EM-D4 phase separated after only one day of storage.

**Figure 3:**
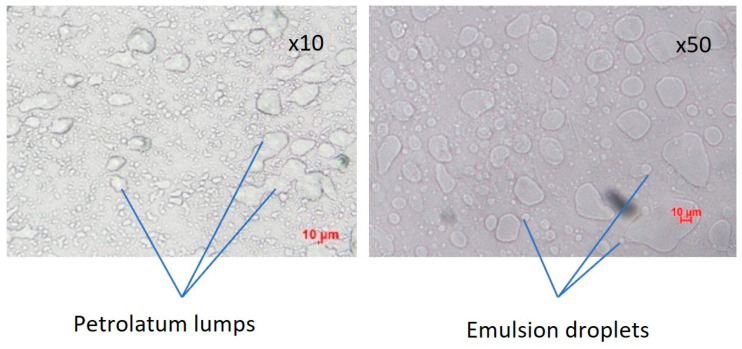
Microscope images of EM-D1 illustrating how “petrolatum lump” and “emulsion droplets” are identified.

Microstructures of the emulsion cream were analyzed using a polarized light microscope. The microscope images shown in Figure 3 reveal two distinct structures. The bigger ones with irregular shape in the size range of tens of micrometers are believed to be petrolatum lumps while the smaller rounder ones that are less than 10 μm in diameter are referred to as emulsion droplets hereafter. Regrettably, due to limitations in the microscopy technique employed, accurately determining droplet size proved challenging, resulting in significant variations in the measured data despite conducting at least 450 measurements for each sample. Consequently, we have opted not to include droplet size as a critical quality attribute in the subsequent experiments.

Oscillatory rheology measurements were performed to study the viscoelastic behavior of the emulsion creams. The visco-elastic parameters obtained for EM-D1 to EM-D4 are shown in Table 3. At the same mineral oil content, increasing emulsifying wax content increased both the complex shear modulus and crossover stress of the emulsion creams when comparing EM-D1 with EM-D3 and EM-D2 with EM-D4. On the other hand, at the same emulsifying wax content, increase in mineral oil content led to a decrease in the complex shear modulus and crossover stress when comparing EM-D1 with DM-D2 and EM-D3 with EM-D4. Emulsifying wax was seen to have a greater impact on the viscoelastic properties than mineral oil. This is probably because emulsifying wax crystallizes out in the final product, providing the product with a more rigid structure. However, higher complex shear modulus and crossover stress did not correlate to improved stability. Despite exhibiting the second highest values for the visco-elastic parameters, EM-D4 showed the highest instability index and phase separation after just one day of storage at 40 °C/75%RH. A closer examination of the instability indices of the four emulsion creams shows that instability index only increased slightly when emulsifying wax content was increased but increased significantly when mineral oil content was increased. These observations suggest that a lower mineral oil content is beneficial towards achieving an emulsion cream that is more physically stable and resistant to shear damage. Based on the above analysis, EM-D1 and EM-D3 were considered the possible candidates for the next DoE study. Since EM-D1 phase separated after one day of storage at 40 °C/75%RH, although it showed a lower instability index compared to EM-D3, we believe that EM-D1 possibly lies at the edge of failure and may be more sensitive to changes in processing parameters. Therefore, EM-D1 composition was chosen for the subsequent DoE studies. The assumption being if we study a less stable composition, we can delineate the process parameter range for stable emulsion in contrast to edges of failure. Choosing a highly stable formulation like EM-D3 might lead to the mistaken conclusion that process parameters have no influence on CQA from a design space perspective.

The subsequent set of experiments (EM-D6 to EM-12) was designed based on EM-D1 composition to investigate if the properties of the emulsion cream can be shifted to the desirable range by altering step 3 homogenization speed [S_A_] and transcutol addition temperature [T_B_]. Homogenization speeds at steps 4 and 5 ([S_B_] and [S_C_]) were fixed at 2500 rpm. During the emulsification stage, 5 min of homogenization [t_B_] was applied twice, first application at ~56 °C and second application at ~50 °C. Transcutol was added at temperature [T_B_] and homogenized for 15 min at 2500 rpm. The operating conditions of EM-D6 to EM-D12 are shown in Table 3. The homogenization speed [S_A_] and transcutol addition temperature [T_B_] did not seem to have any significant effect on the rheological properties G* and σ_cross_ (EM-D1 vs. EM-D8, EM-D6 vs. EM-D9, and EM-D7 vs. EM-10). Most importantly, EM-D6 to EM-D10 exhibited similar stability as assessed by the Instability Index measured by Lumisizer. Since changing the operating conditions did not improve stability of the emulsion cream, we suspected that the composition required further adjustment. In the subsequent two runs (EM-D11 and EM-D12), petrolatum content was increased from 10 wt% to 20 wt% in the hope that the stability of the emulsion creams could be enhanced by the increased viscosity. For these runs, the homogenization speed [S_A_] was set at 8000 rpm, which is the same as used in the first set of experiments EM-D1 to EM-D4. Transcutol was added at two temperatures [T_B_], 35 °C and 43 °C. With the increase in white petrolatum content, G* of the emulsion cream increased almost two-fold but σ_cross_ remained almost the same (EM-D8 vs. EM-D11, EM-D1 vs. EM-D12). Unfortunately, IS increased significantly which means that the increase in white petrolatum content worsened the stability of the emulsion cream even though G* increased. This second set of experiments showed that the stability of the emulsion cream based on EM-D1 composition cannot be improved further. This may be due to the inherently unstable nature of the EM-D1 emulsion. In fact, the characterization of the emulsion cream was challenging due to the unstable nature of the emulsion cream which posed some uncertainty to the data acquired since the emulsion cream may be phase separating during measurement. Thus, EM-D3 composition, which is a more stable system (higher emulsifier and low oil content), was selected for subsequent process DoE studies.

EM-D13 process studies were conducted using the same composition and operating conditions as EM-D3, except that the emulsification stage step 3 homogenization speed [S_A_] was lowered to 2500 rpm. The homogenization speed [S_A_] did not have any significant effect on the rheological properties and stability as seen from the similar G*, σ_cross_, and IS as EM-D3 (Table 4). EM-D3N1 and EM-D3N2 (based on EM-D3 composition) were further conducted to investigate the effect of homogenization duration by increasing the duration of the various homogenization steps. Step 3 homogenization [S_A_] and step 4 homogenization [S_B_] were both increased from 10 to 30 min; step 5 homogenization [S_C_] was increased from 15 to 30 min, and step 6 homogenization was also increased from 5 to 10 min. The operating conditions of EM-D3N1 and EM-D3N2 are shown in Table 4. Even with the increase in homogenization durations, the measured rheological properties and IS remained relatively unchanged. This indicates that step 3 homogenization speed [S_A_] and the duration of the different homogenization steps are not the critical processing parameters.

The assumption in the previous DoE studies was that the properties of the emulsion were dependent upon the processing steps required to disperse the internal phase; however, petrolatum and the emulsifying wax components are melted and, upon cooling, could form the semi-solid to solid phases, the distribution of which could dictate the microstructure and properties. Thus, it appeared that the critical processing steps could be after the emulsification steps, so in the next and, also, the final DoE, we decided to focus on the steps after transcutol addition. In the final DoE, an additional shearing step was incorporated during the cooling step to assess if the additional shear would influence the microstructure of the emulsion and, in turn, the stability and properties of the product.

### 3.2. Final Design of Experiment

**Figure 4:**
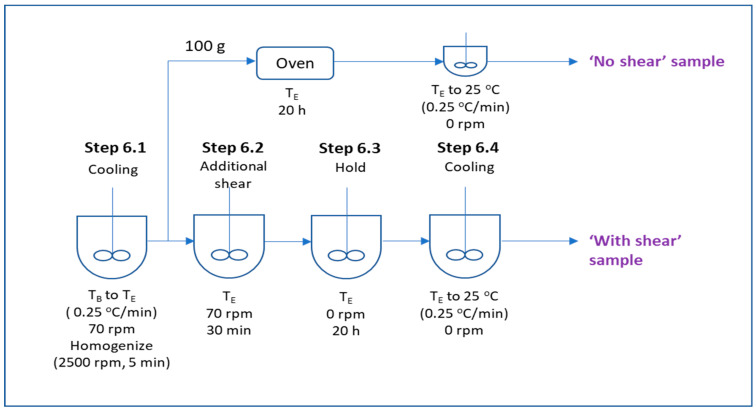
Process flow diagram showing sampling points for ‘no shear’ and ‘with shear’ samples.

In the final DoE, the effect of the additional shear and the temperature at which the shear was applied during the cooling stage was studied. The process flow for the incorporation of the additional shear is shown in Figure 1b. Besides the additional shear step, an additional 20 h of holding time was added to the process from the time when the emulsion reached the temperature [T_E_] to the final cooling stage. Even though previous experiments suggested that step 3 homogenization is not the critical processing parameter, two levels of step 3 homogenization speed [S_A_] (8000 rpm and 2500 rpm) were assessed since a lower speed would be preferred in the actual manufacturing process. The transcutol addition temperature [T_B_] was set at 43 °C, and homogenization speeds [S_B_] and [S_C_] were set at 2500 rpm. The cooling rate from 43 °C to temperature [T_E_] was set at 0.25 °C/min, and the temperature [T_E_] used in this study were 35, 30, and 25 °C. Approximately 100 g of sample (sample denoted with suffix “-NS”) was collected at step 6.1 from the vessel once the temperature reached [T_E_] and stored in an oven at temperature [T_E_] for 20 h. The sample was analyzed after cooling to 25 °C at 0.25 °C/min. Additional shear was applied to the remaining emulsion cream in the vessel using a paddle mixer at 70 rpm for 30 min (step 6.2). The paddle mixer was then switched off, and the emulsion cream was left in the vessel at temperature [T_E_] for 20 h (step 6.3). After 20 h, the remaining cream was cooled from [T_E_] to 25 °C at 0.25 °C/min (step 6.4), and a sample was collected for characterization and named with a suffix “-S”. The operating conditions of the final DoE are shown in Table 5. The schematic illustrating the workflow and sample collection points after step 5 is shown in Figure 4.

#### 3.2.1. Stability of Emulsion

**Figure 5:**
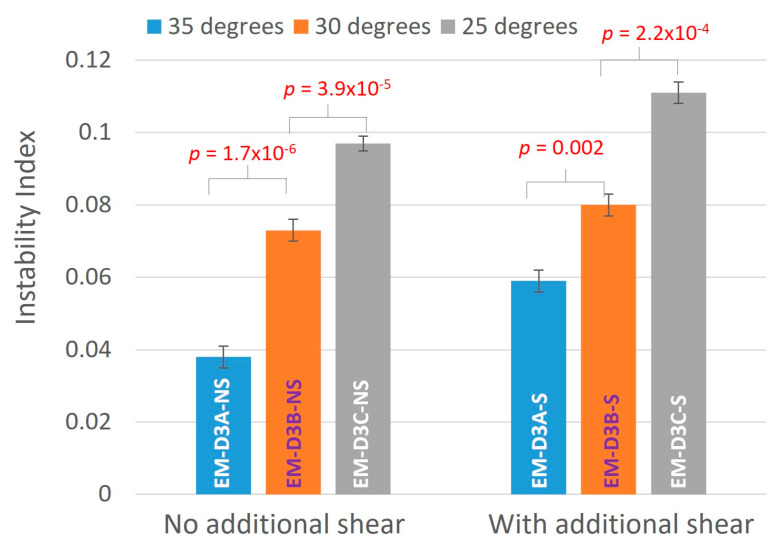
Instability index of EM-D3A, EM-D3B, and EM-D3C. The temperature values in the legend refer to the holding temperature [T_E_].

The instability indices of the emulsion cream produced in the final DoE are shown in Table 5 and Figure 5. Consistent with the previous experiments, the effect of the stage 3 homogenization speed [S_A_] was not significant, although the instability indices of the samples prepared at S_A_ = 8000 rpm were slightly lower than those prepared at 2500 rpm. This suggests that the actual manufacturing process can be safely conducted at stage 3’s homogenization speed of 2500 rpm. Comparing samples prepared at the same T_E_, additional shear adversely impacted the stability of the emulsion cream as seen from the higher instability indices from the samples that were subjected to additional shear compared to those without additional shear. From Figure 5, it can also be seen that the stability of the emulsion was improved if the additional shear was applied at a higher T_E_. Thus, the results suggest that emulsion cream processed at a higher temperature [T_E_] and without additional shearing is favorable. The addition of a holding period during the cooling stage after transcutol addition at 35 °C is advantageous towards achieving a more stable emulsion cream compared to the original process (direct cooling from 43 °C to 25 °C). ANOVA analysis confirmed that the instability index data are statistically significantly different between the different samples (*p* < 0.05).

#### 3.2.2. Rheological Properties of Emulsion Cream

**Figure 6:**
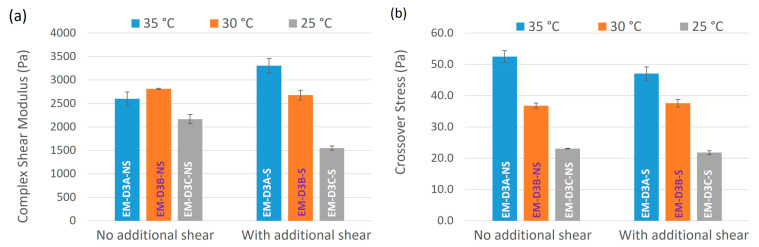
Effect of additional shear (70 rpm and 30 min) and temperature [T_E_] on (**a**) complex shear modulus and (**b**) crossover stress. The temperature values in the legend refer to the holding temperature [T_E_].

**Figure 7:**
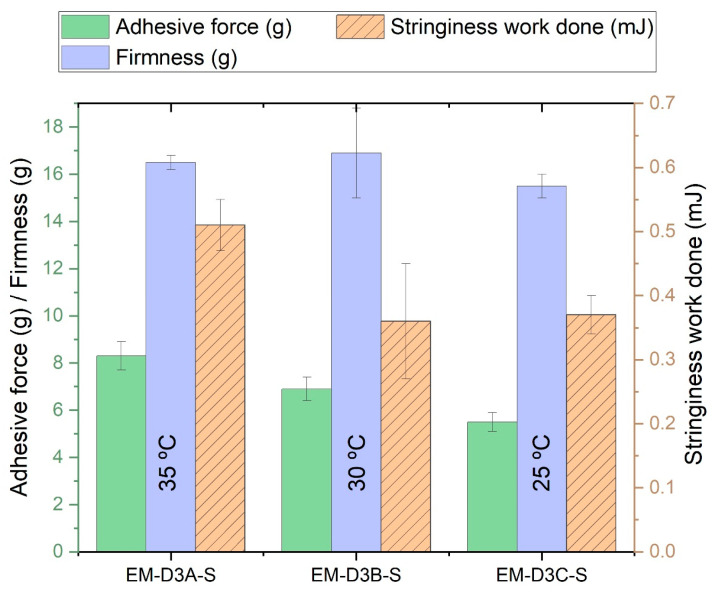
Texture analysis results of samples EM-D3A-S, EM-D3B-S, and EM-D3C-S prepared with holding temperature at 35 °C, 30 °C, and 25 °C, respectively.

Complex shear moduli and crossover stresses of the samples from the final DoE obtained from oscillatory strain sweep experiments are shown in Table 5 and graphically represented in Figure 6. Only data obtained from S_A_ = 8000 rpm are plotted in Figure 7 since the data followed the same trend at 2500 rpm. In general, samples subjected to additional shear exhibited a lower complex shear modulus compared to those not subjected to additional shear at the same temperature [T_E_] except for EM-D3A-NS processed at 35 °C (Figure 6a). The same trend was observed for crossover stress in Figure 6b with an outlier of the EM-D3B-NS sample. The outlier points could be due to measurement error such as slippage on geometry plate. When we compared the samples processed at different holding temperature [T_E_], both complex shear modulus and crossover stress were seen to decrease with decreasing temperature [T_E_]. The only exception was EM-D3B-NS which showed a higher complex shear modulus than EN-D3A-NS. Overall, the oscillatory strain sweep results suggest that emulsion cream processed at a higher holding temperature and without being subjected to additional shear exhibit a more defined semisolid microstructure.

#### 3.2.3. Sensorial Properties of Emulsion Cream

Textural analysis results are shown in Table S1. The results for the samples obtained at S_A_ = 8000 rpm are also graphically shown in Figure 7. It is clear from the plot that adhesive force decreased with the holding temperature [T_E_], but the trends for firmness and stringiness were not obvious. Therefore, ANOVA analysis was performed on the data, and the calculated *p*-values are shown in Figures S2–S4. The *p*-values for adhesiveness are <0.05, confirming that there were significant differences between samples prepared at different holding temperatures. The *p*-values for stringiness work done are all >0.05 which indicate that holding temperature [T_E_] did not have any significant effect on stringiness work done. However, the trend for firmness is less straightforward, but there appears to be a transition temperature at which firmness decreases, but this transition temperature varies with the step 3 homogenization speed [S_A_]. Textural analysis results suggest that it is possible to obtain the desired firmness, stringiness, and adhesiveness of the emulsion cream by adjusting the processing parameters.

**Figure 8:**
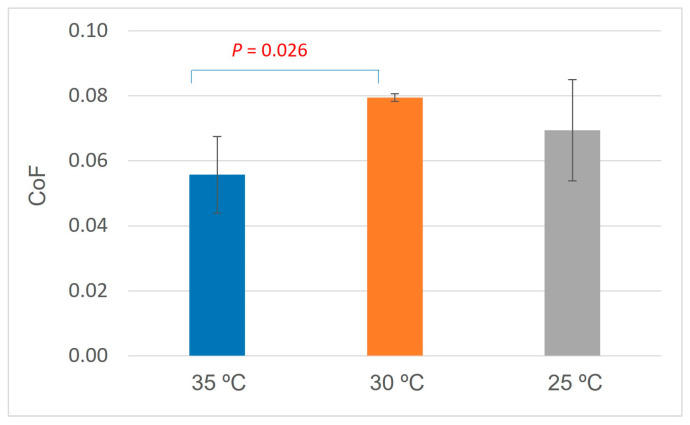
Friction coefficient of samples EM-D3A-S, EM-D3B-S, and EM-D3C-S prepared with holding temperature [T_E_] at 35 °C, 30 °C, and 25 °C, respectively.

The average friction coefficient (CoF) for EM-D3B-S is the highest, followed by EM-D3C-S, and EM-D3A-S (Figure 8). The difference between EM-D3A-S and EM-D3B-S is statistically significant with *p* value of 0.026. The smallest friction coefficient result is observed from the emulsion sample prepared at holding temperature of 35 °C before cooling down to 25 °C. It is worth noting that the EM-D3A-S sample is also the sample with the largest elasticity observed from the rheology data in the linear viscoelastic regime (LVER). It seems that the holding temperature [T_E_] of 35 °C not only provided the strongest bulk structure but also facilitated a lubricative effect of the cream.

#### 3.2.4. In Vitro Release of Emulsion Cream

**Figure 9:**
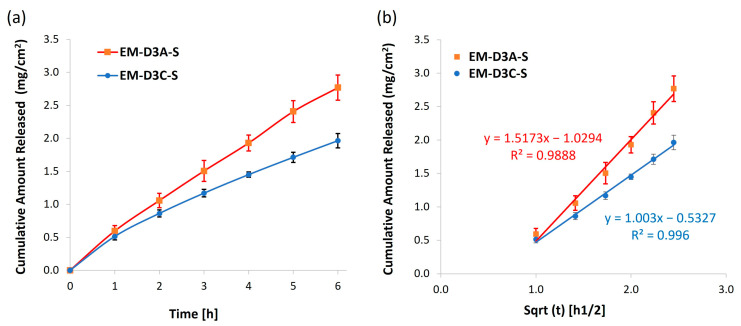
(**a**) Cumulative release of lidocaine from ointments EM-D3A-S and EM-D3C-S; (**b**) Cumulative release data fitted to the Higuchi model.

In vitro release studies were performed using the most stable (EM-D3A-S) and least stable (EM-D3C-S) emulsion cream processed with additional shear in the final DoE to investigate if the stability of the emulsion cream would affect the drug release performance. Lidocaine was used as the model drug for this study. Figure 9 shows the release profiles of lidocaine from EM-D3A-S and EM-D3C-S, with both containing the same loading of lidocaine (1 wt%). The cumulative release of lidocaine from EM-D3A-S was higher than from EM-D3C-S. This shows that additional shear applied at 35 °C enhanced both the stability and the active release performance.

#### 3.2.5. Stabilization Mechanism

**Figure 10:**
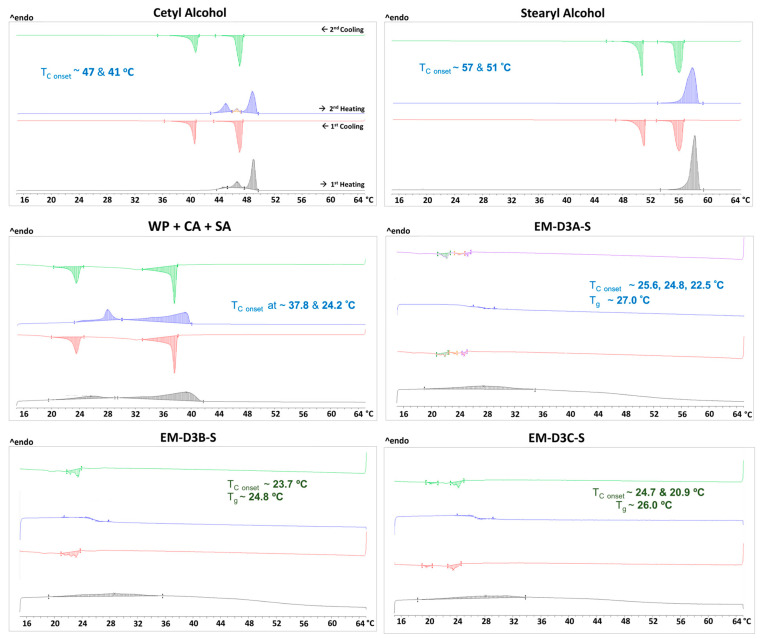
DSC thermograms of cetyl alcohol (CA), stearyl alcohol (SA), combinations of cetyl alcohol, stearyl alcohol, and white petrolatum (WP), and the three emulsion creams (EM-D3A-S, EM-D3B-S, EM-D3C-S).

Our experimental results showed that the modified process with the additional holding time in the middle of the cooling stage from 43 °C to 25 °C enhanced the stability of the emulsion cream significantly (reduction in instability index measured by Lumisizer) compared to the original process. The temperature at which the cooling was paused and the additional shear was applied play a critical role in the stability of the emulsion cream. Out of the three temperatures studied, 35 °C yielded the most stable cream followed by 30 °C and 25 °C. To explain this observation, we performed DSC experiments to analyze if the stabilization of the cream could be related to any phase transition behavior. The DSC thermograms of the pure components, combinations of components, and the actual cream formulations are shown in Figure 10. White petrolatum only underwent glass transition [6], while GMS (Figure S5) did not exhibit any thermal event. Both cetyl alcohol and stearyl alcohol showed two crystallization peaks: 47 and 41 °C for CA and 57 and 51 °C for SA. These crystallization temperatures agree well with the values reported in the literature [21,22]. The first exothermic peak was ascribed to the transition from the liquid to solid phase, and the second exothermic peak was ascribed to the transformation from the hexagonal to orthorhombic structure [21,22]. When CA and SA were mixed with WP at the same proportion as the composition of the emulsion cream, the crystallization peaks of the fatty alcohols (CA and SA) were shifted to lower temperatures of 37.8 °C and 24.2 °C. In the cream formulations, the crystallization onset temperatures were further shifted to below 30 °C. In addition, glass transition was observed between 25 and 27 °C for the three emulsion creams. Since the mixtures are agitated and homogenized during the actual emulsion cream preparation process, the crystallization of the fatty alcohols is likely to occur at a higher temperature than that observed under the static condition of the DSC experiments. This is because agitation will lower the activation energy required for crystal nucleation, which in turn leads to faster and earlier nucleation [23,24,25,26,27].

**Figure 11:**
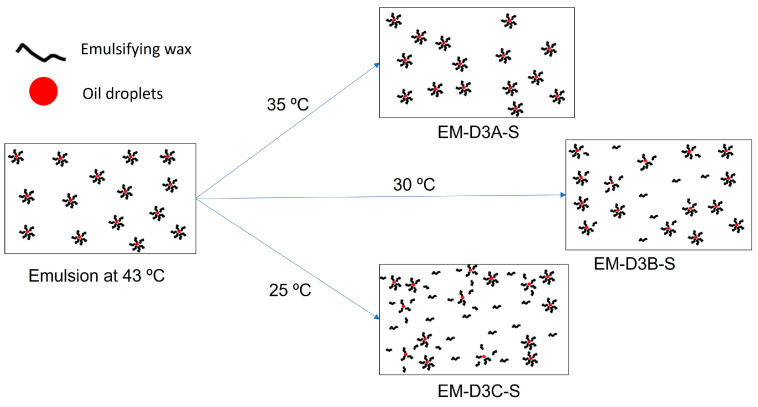
Proposed mechanism for the stabilization of the emulsion by emulsifying wax when shear is applied at different holding temperatures. At 35 °C, emulsifying wax molecules are mobile enough to rebuild the microstructure even when shear is applied. At 30 °C, some of the emulsifying wax molecules start to crystallize and the emulsion is becoming viscous which hinders the reconstruction of the microstructure once it is disrupted by shear. At 25 °C, the emulsion is even more viscous so additional shear will result in more severe damage to the microstructure. Therefore, the stability of the emulsion decreases with decrease in holding temperature [T_E_].

Based on the DSC results, we propose the hypothesis as illustrated in Figure 11 to explain the stability mechanism for holding at 35 °C compared to lower temperatures. When transcutol is added at 43 °C followed by homogenization, oil droplets are surrounded by emulsifying wax, and the emulsion is freely flowable. When the emulsion is cooled to 35 °C, the fatty alcohols stay in a “liquid state” and surround the oil droplets serving as the emulsifiers. The emulsion is still flowable with a relatively low viscosity at this temperature so the application of shear will not disrupt the overall microstructure and disrupt the emulsifier layer around the droplets, since the components are mobile enough to rebuild the emulsion structure during the 20 h holding time. When the emulsion is finally cooled to 25 °C without agitation, the fatty alcohols start to crystallize, the emulsion cream turns glassy, and the microstructure built earlier becomes “frozen”. However, if the emulsion is cooled to 30 °C, some of the fatty alcohols would have started to crystallize out. The emulsion is more viscous than at 35 °C, and the microstructure may not be rebuilt once disturbed by agitation. Some of the crystallized fatty alcohols may be distributed in the aqueous phase rather than serving as a flexible emulsifier film around the oil droplets. Upon further cooling to 25 °C, the final emulsion cream will contain discrete oil droplets not emulsified by emulsifiers, and that explains the lower stability observed compared to 35 °C and if the shear was applied at 35 °C. The disruption to the microstructure will be more severe for the case of EM-D3C-S since the emulsion cream is significantly more viscous at 25 °C as it is below the glass transition temperature of the emulsion cream. Samples not subjected to additional shear show higher stability compared to those with shear, because shear tends to disrupt/destroy some of the microstructure built.

The additional holding time halfway during the cooling process (stress relaxation) is also seen to be advantageous to improving the stability of the emulsion cream. The holding time allows the emulsion microstructure to be built up more completely before the final cooling step “freezes” the microstructure. The holding temperature above 30 °C is more effective due to high molecular mobility and, hence, more flexible film and stable emulsion formation.

## 4. Conclusions

This study demonstrated the importance of composition and process parameters on the critical quality attributes of emulsion cream products. In the preliminary experiments, the stability of an inherently unstable emulsion composition could not be improved by changing the processing parameters, and all the resultant emulsion creams phase separated within a day at 40 °C/75%RH. When a suitably stable emulsion composition was used in the subsequent experimental studies based on the original manufacturing process, all the processing parameters did not show any significant effect on the stability of the emulsion cream as indicated by the instability index. As a result, the final DoE focused on the processing steps after the transcutol addition and emulsification steps by incorporating a holding period halfway through the cooling process and an additional shearing step during the holding temperature (T_E_, step 6.1). The overall results of the final emulsion DoE suggested that the addition of a holding period halfway through the cooling stage after transcutol addition is advantageous towards achieving a more stable emulsion cream compared to direct cooling from 43 °C to 25 °C. The additional holding period allows time for more stable and complete emulsion formation and microstructure build-up. In the range of holding temperature studied (25–35 °C), the higher the holding temperature, the higher the stability of the emulsion cream. The application of shear (via paddle mixer) during the holding period adversely impacted the stability of the emulsion cream compared to those without additional shear due to the disruption of the emulsion microstructure. IVRT studies also showed that the release of lidocaine from the most stable emulsion produced with a holding temperature of 35 °C is higher than from the least stable emulsion produced with a holding temperature of 25 °C. This shows that the emulsion prepared with the holding temperature of 35 °C enhanced both the stability and the active release performance.

## Figures and Tables

**Table 1:** Compositions of emulsion creams.

Ingredient	Phase	Wt.%
Purified water (q.s.)	Aqueous phase	38.0–48.0
PEG400		15.0
Light mineral oil	Oil phase	5.0–10.0
White petrolatum (WP)		10.0
Oleyl alcohol		2.0
Emulsifying wax (GMS, Tween20, CA, SA)	Emulsifier	5.0–10.0
Transcutol	Active Solvent	15.00
Total		100

**Table 2:** Levels of each processing condition studied.

	**Processing Conditions**	**Levels**
[S_A_]	Step 3 homogenization speed	2500, 4000, 8000 rpm
[t_A_]	Step 3 homogenization duration	10, 30 min
[T_B_]	Transcutol addition temperature	35 ± 3, 43 ± 3 °C
[S_B_]	Step 4 homogenization speed	2500 rpm
[t_B_]	Step 4 homogenization duration	5 min (twice, at ~56 °C and 50 °C) or 30 min (once, at 60 °C)
[S_C_]	Step 5 homogenization speed	2500 rpm
[t_C_]	Step 5 homogenization duration	15, 30 min
[T_E_]	Additional shearing temperature during the final hold and cooling stage	35 ± 3, 30 ± 3, 25 ± 3 °C

**Table 3:** Operating conditions, Lumisizer instability index, droplet size, and visco-elastic properties for dry runs EM-D1 to DM-12.

Processing Stages	Conditions	Sample										
		EM-D1	EM-D2	EM-D3	EM-D4	EM-D6	EM-D7	EM-D8	EM-D9	EM-D10	EM-D11	EM-D12
Step 3 mixing	Homogenization speed [S_A_] (rpm)	8000	8000	8000	8000	2500	4000	8000	2500	4000	8000	8000
	Homogenization duration [t_A_] (min)	10	10	10	10	10	10	10	10	10	10	10
Transcutol addition	Temperature [T_B_] (°C)	43	43	43	43	43	43	35	35	35	35	43
Step 4 cooling from 60 °C to [T_B_]	Homogenization speed [S_B_] (rpm)	2500	2500	2500	2500	2500	2500	2500	2500	2500	2500	2500
Step 5 mixing after transcutol addition	Homogenization speed [S_C_] (rpm)	2500	2500	2500	2500	2500	2500	2500	2500	2500	2500	2500
Composition	Mineral oil (wt%)	5	10	5	10	5	5	5	5	5	5	5
	Emulsifying wax (wt%)	5	5	10	10	5	5	5	5	5	5	5
	White Petrolatum (wt%)	10	10	10	10	10	10	10	10	10	20	20
Microscopy measurement	Droplet size (µm)	7.70 ± 9.35	N.M.	6.92 ± 6.15	N.M.	7.86 ± 8.53	6.63 ± 6.58	6.60 ± 8.22	7.53 ± 7.55	7.03 ± 7.62	8.24 ± 10.67	7.36 ± 11.31
Stability	Bottle test @40 °C/75%RH (1 Day)	PS	PS	No PS	PS	PS	PS	PS	PS	PS	PS	PS
	Instability Index (IS)	0.090 ± 0.005	0.151 ± 0.005	0.101 ± 0.004	0.166 ± 0.001	0.112 ± 0.001	0.100 ± 0.002	0.096 ± 0.004	0.101 ± 0.004	0.102 ± 0.002	0.134 ± 0.001	0.142 ± 0.002
Complex Shear Modulus (G*) (Pa)		688 ± 30	606 ± 2.7	1867 ± 93	1607 ± 43	823 ± 47	696 ± 16	698 ± 23	600 ± 42	557 ± 21	1178 ± 235	1063 ± 93
Crossover Stress (σ_cross_) (Pa)		15.0 ± 1.1	8.80 ± 1.3	24.4 ± 0.9	18.4 ± 0.5	11.9 ± 0.2	13.5 ± 0.3	15.3 ± 0.2	13.5 ± 1.4	14.0 ± 0.4	16.1 ± 1.5	13.7 ± 2.2

PS: phase separation, N.M.: not measured.

**Table 4:** Operating conditions, Lumisizer instability index, and visco-elastic properties for dry runs EM-D3, EM-D13, EMD3N1, and EM-D3N2.

Processing Stages	Conditions	Sample			
		EM-D13	EM-D3	EM-D3N1	EM-D3N2
Step 3 mixing	Homogenization speed [S_A_] (rpm)	2500	8000	8000	2500
	Homogenization duration [t_A_] (min)	10	10	30	30
Transcutol addition	Temperature [T_B_] (°C)	43	43	43	43
Step 4 cooling from 60 °C to [T_B_]	Homogenization speed [S_B_] (rpm)	2500	2500	2500	2500
	Homogenization Duration [t_B_] (min)	10	10	30	30
Step 5 mixing after transcutol addition	Homogenization speed [S_C_] (rpm)	2500	2500	2500	2500
	Homogenization duration [t_C_](min)	15	15	30	30
Composition	Mineral Oil Content (wt%)	5	5	5	5
	Emulsifier Content (wt%)	10	10	10	10
	White Petrolatum (wt%)	10	10	10	10
Stability	Bottle test @40 °C/75%RH (1-Day)	No PS	No PS	No PS	No PS
	Instability Index (IS)	0.101 ± 0.002	0.101 ± 0.004	0.106 ± 0.001	0.110 ± 0.001
Complex Shear Modulus (G*) (Pa)		1831 ± 112	1867 ± 93	1982 ± 122	1814 ± 16
Crossover Stress (σ_cross_) (Pa)		23.7 ± 2.6	24.4 ± 0.9	25.4 ± 2.3	23.6 ± 0.2

PS: phase separation.

**Table 5:** Operating conditions, Lumisizer instability index, and viscoelastic properties for dry runs EM-D3A, EM-D3B, and EM-D3C.

Processing Stages	Conditions	Run Number/Sample												
		Run 1	Run 2	Run 3	Run 4	Run 5	Run 6	Run 7	Run 8	Run 9	Run 10	Run 11	Run 12	Run 13
		EM-D3	EM-D3A-NS	EM-D3A1-NS	EM-D3A-S	EM-D3A1-S	EM-D3B-NS	EM-D3B1-NS	EM-D3B-S	EM-D3B1-S	EM-D3C-NS	EM-D3C1-NS	EM-D3C-S	EM-D3C1-S
Step 3 mixing	[S_A_] (rpm)	8000	8000	2500	8000	2500	8000	2500	8000	2500	8000	2500	8000	2500
	[t_A_] (min)	10	10	10	10	10	10	10	10	10	10	10	10	10
Transcutol addition	[T_B_] (°C)	43	43	43	43	43	43	43	43	43	43	43	43	43
Step 4 cooling	[S_B_] (rpm)	2500	2500	2500	2500	2500	2500	2500	2500	2500	2500	2500	2500	2500
	[t_B_] (min)	10	10	10	10	10	10	10	10	10	10	10	10	10
Step 5 mixing	[S_C_] (rpm)	2500	2500	2500	2500	2500	2500	2500	2500	2500	2500	2500	2500	2500
	[t_C_] (min)	15	15	15	15	15	15	15	15	15	15	15	15	15
Holding temperature	[T_E_] (°C)	-	35	35	35	35	30	30	30	30	25	25	25	25
Additional shear	Mixing speed (rpm)	-	-	-	70	70	-	-	70	70	-	-	70	70
	Duration (min)	-	-	-	30	30	-	-	30	30	-	-	30	30
Stability	Bottle test @40 °C/75%RH (1-Day)	No PS	No PS	No PS	No PS	No PS	No PS	No PS	No PS	No PS	No PS	No PS	No PS	No PS
	Instability Index (IS)	0.101 ± 0.004	0.038 ± 0.003	0.051 ± 0.003	0.059 ± 0.003	0.071 ± 0.001	0.073 ± 0.003	0.084 ± 0.007	0.080 ± 0.003	0.092 ± 0.005	0.097 ± 0.002	0.103 ± 0.008	0.111 ± 0.003	0.114 ± 0.003
Complex Shear Modulus (G*) (Pa)		1867 ± 93	2598 ± 149	3210 ± 146	3308 ± 156	2878 ± 51	2811 ± 14	3378 ± 127	2677 ± 103	2831 ± 161	2167 ± 99	2412 ± 42	1549 ± 44	1863 ± 42
Crossover Stress (σ_cross_) (Pa)		24.4 ± 0.9	52.5 ± 1.9	50.0 ± 0.9	47.1 ± 2.1	51.3 ± 2.0	36.8 ± 0.8	31.6 ± 0.7	37.6 ± 1.2	28.7 ± 1.3	23.1 ± 0.1	28.3 ± 0.4	21.8 ± 0.6	24.6 ± 1.6

PS: phase separation, S: homogenization speed, t: time, T: temperature.

## Data Availability

The data that support the findings of this study are available from the corresponding author, Ron Tau Yee Lim (lim_tau_yee@isce2.a-star.edu.sg) upon reasonable request.

## Supplementary Materials

The following supporting information can be downloaded at: https://www.mdpi.com/article/10.3390/pharmaceutics16060773/s1, Figure S1. Bottle tests of EM-D1 to EM-D4 at 40 °C/75%RH; Figure S2: Adhesiveness values of emulsion cream samples prepared at different holding temperature; Figure S3: Stringiness work done values of emulsion cream samples prepared at different holding temperatures; Figure S4: Firmness values of emulsion cream samples prepared at different holding temperatures; Figure S5: DSC thermogram of glyceryl monostearate; Table S1. Sensorial properties of emulsion cream samples.
