# Supplementary material for: The Effect of Process Parameters on the Microstructure, Stability, and Sensorial Properties of an Emulsion Cream Formulation
Source: Pharmaceutics. 2024 Jun 6;16(6):773. doi: 10.3390/pharmaceutics16060773 (PMC11207101; doi:10.3390/pharmaceutics16060773)
Supplement: Supplementary file 1 [file pharmaceutics-16-00773-s001.zip › pharmaceutics-3000418-supplementary.pdf]

Supplementary Information

Effect of Processing Parameters on the Microstructure, Stability, and Sensorial Properties of an Emulsion Cream Formulation

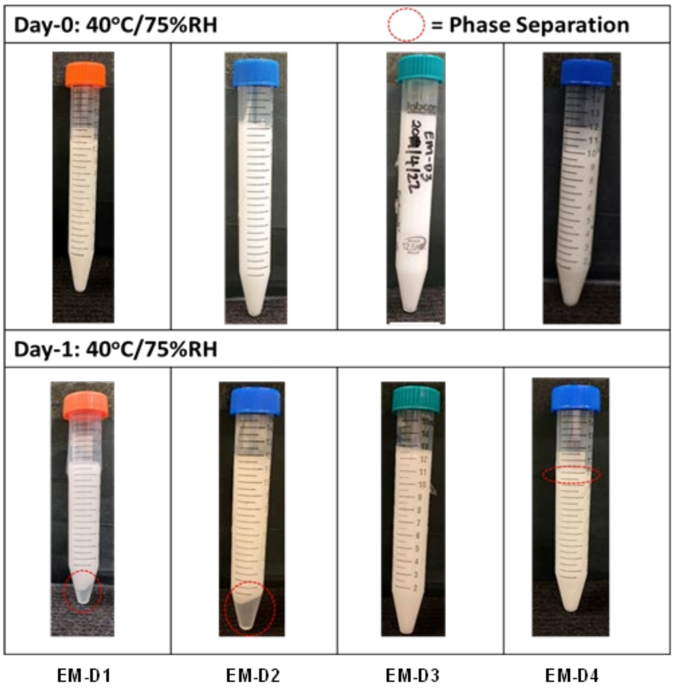

Figure S1. Bottle tests of EM-D1 to EM-D4 at 40ºC/75%RH

Table S1. Sensorial properties of emulsion cream samples

Sample	Instability Index (IS)	Firmness (g)	Adhesive force (g)	Stringiness work done (mJ)	Friction coefficient (CoF)
EM-D3A-S	0.059 ± 0.003	16.5 ± 0.3	8.3 ± 0.6	0.51 ± 0.04	0.056 ± 0.012
EM-D3A1-S	0.071 ± 0.003	13.0 ± 0.6	6.4 ± 0.2	0.36 ± 0.03	0.050 ± 0.019
EM-D3B-S	0.080 ± 0.003	16.9 ± 1.9	6.9 ± 0.5	0.36 ± 0.09	0.079 ± 0.001
EM-D3B1-S	0.092 ± 0.005	9.87 ± 0.4	5.1 ± 0.2	0.30 ± 0.05	0.066 ± 0.009
EM-D3C-S	0.111 ± 0.003	15.5 ± 0.5	5.5 ± 0.4	0.37 ± 0.03	0.069 ± 0.016
EM-D3C1-S	0.114 ± 0.003	10.2 ± 0.7	4.4 ± 0.3	0.29 ± 0.01	0.056 ± 0.008

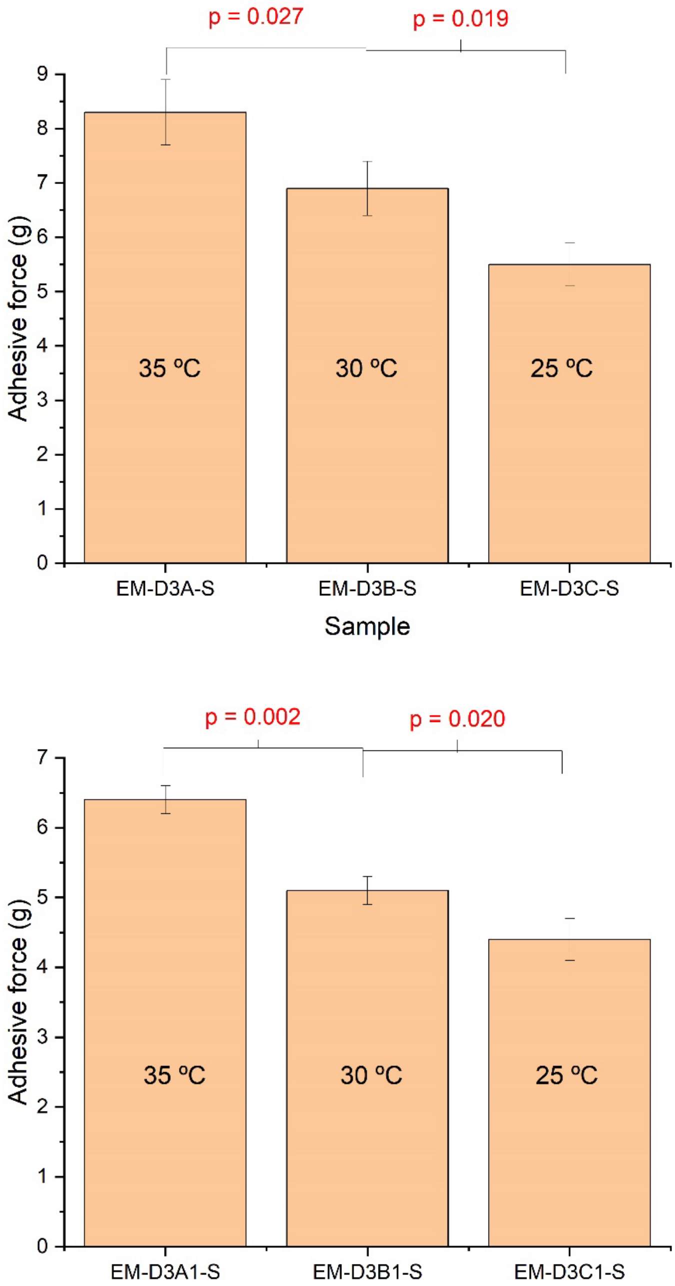

Figure S2. Adhesiveness values of emulsion cream samples prepared at different holding temperature.

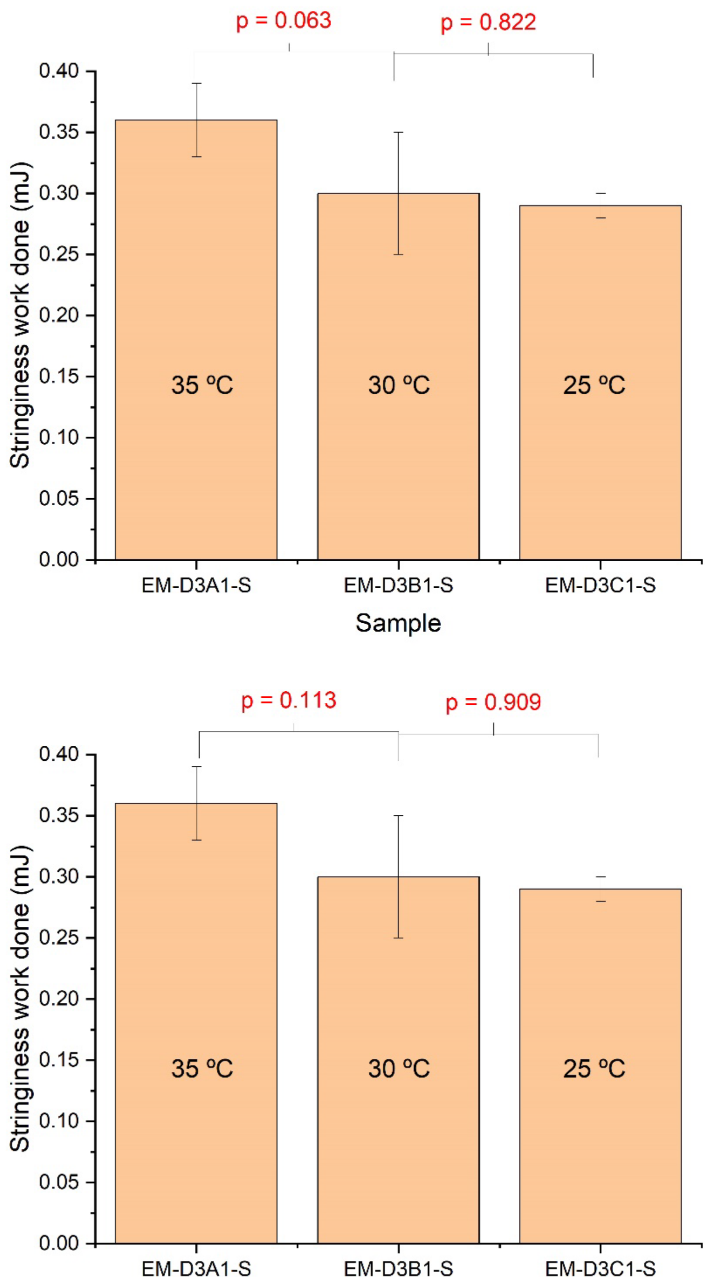

Figure S3. Stringiness work done values of emulsion cream samples prepared at different holding temperatures.

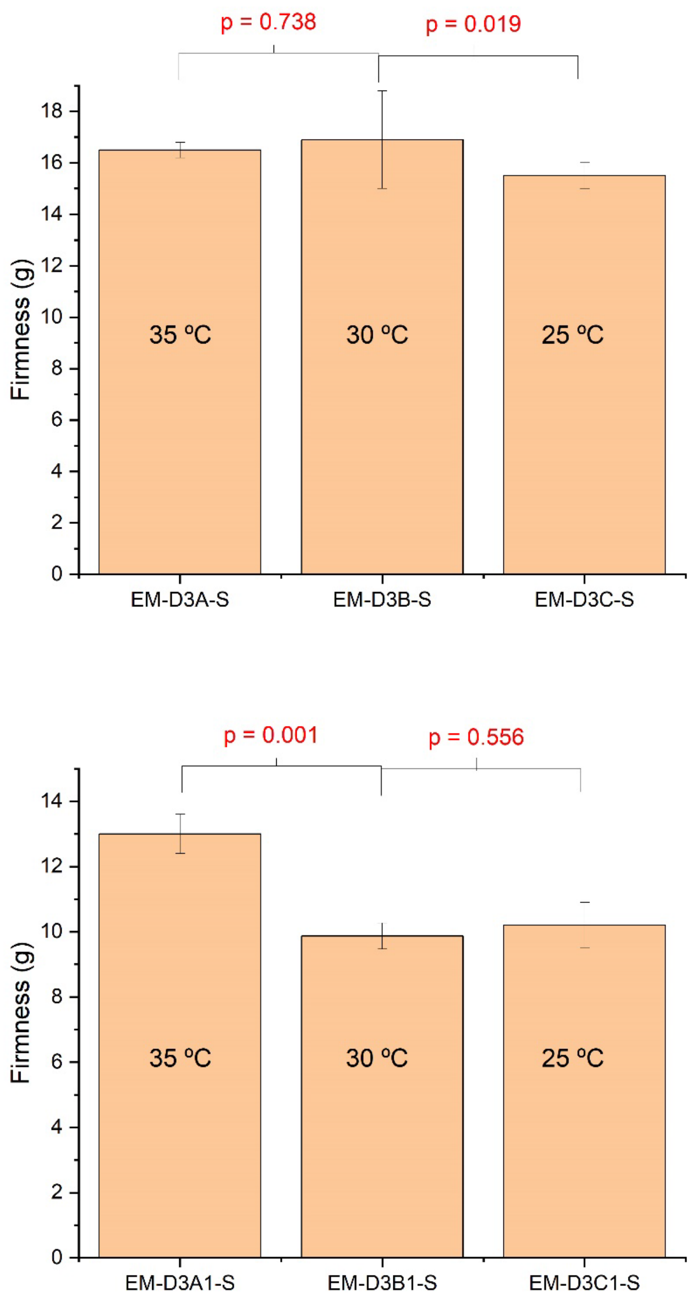

Figure S4. Firmness values of emulsion cream samples prepared at different holding temperatures.

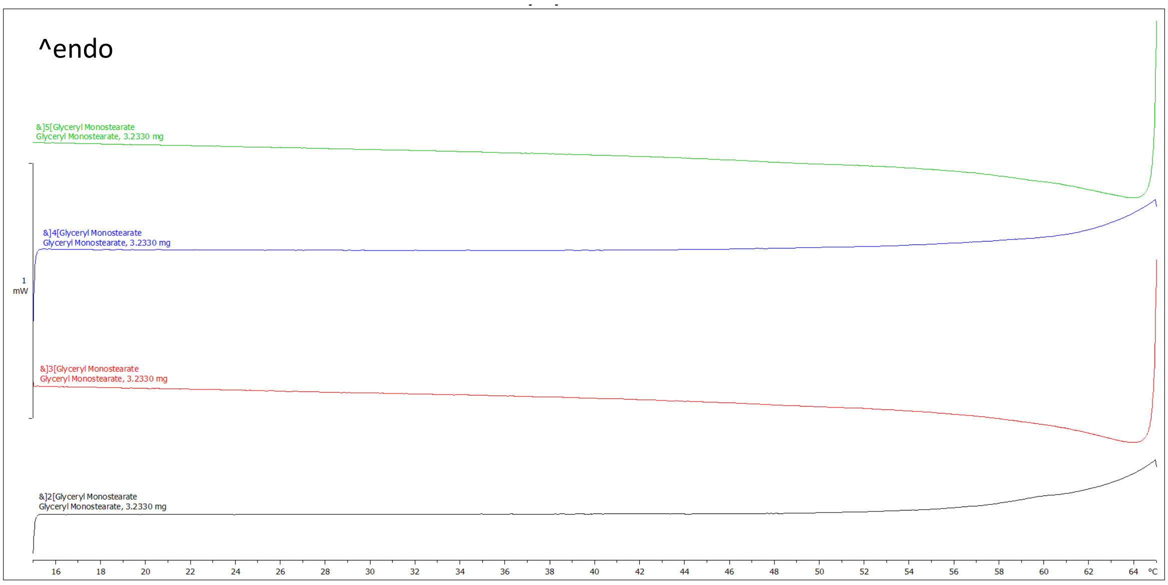

Figure S5. DSC thermogram of glyceryl monostearate.
